# Diversified crop rotation reshapes soil nutrients, microbial communities, and functions in sorghum fields

**DOI:** 10.3389/fmicb.2026.1958119

**Published:** 2026-10-27

**Authors:** Manjing Chen, Yuwen Jiang, Jiaqi Shen, Yan Ren, Lengbo Zhou, Chunge Wang, Guilan Chen, Mingbo Shao

**Affiliations:** 1 Institute of Dryland Crops, Guizhou Academy of Agricultural Sciences, Guiyang, China; 2 Chemistry and Biology Teaching and Research Group, The Affiliated High School of Guizhou Academy of Agricultural Sciences, Guiyang, China

**Keywords:** diversified crop rotation, functional prediction, microbial community structure, soil health, sorghum

## Abstract

**Introduction:**

Long-term continuous sorghum cropping degrades soil environments and causes continuous cropping obstacles. This study investigated whether diversified crop rotation can maintain soil health and improve the soil microenvironment for sorghum growth.

**Methods:**

Using continuous sorghum cropping as the control (SSS), three rotation systems were established: sorghum-potato-sorghum (SPS), sorghum-common vetch-sorghum (SVS), and sorghum-green pea shoots-sorghum (SGS). Soil physicochemical properties, microbial community structure and diversity were analyzed using Illumina NovaSeq high-throughput sequencing, and functional traits were predicted using FAPROTAX and FUNGuild. Mantel tests and co-occurrence network analysis were also performed.

**Results:**

All three rotation treatments improved soil physicochemical properties to varying degrees, with SPS showing the greatest nutrient enhancement. SVS markedly increased bacterial richness and diversity, while SPS significantly enhanced fungal richness and diversity. β-diversity analysis revealed that rotation treatments differed significantly from SSS, with SVS and SGS showing similar bacterial structures and SPS and SVS showing similar fungal structures. Functionally important microbial groups increased in rotation soils, whereas the pathogenic genus *Fusarium* remained relatively high in SPS. Mantel tests indicated that soil pH, TN, TP, TK, and AK were key factors influencing microbial community structure, with AK showing the strongest correlation. Nitrogen fixation and cellulose degradation were the dominant bacterial functions, while saprotrophic and ectomycorrhizal fungi were enriched in rotation soils. Co-occurrence network analysis showed that the rotation network had higher average degree, network density, modularity, and a larger connected component than the continuous cropping network.

**Discussion:**

These findings indicate that diversified crop rotation effectively elevates soil nutrient levels, reshapes microbial community structure and ecological functions, and supports high-quality and stable sorghum production.

## 1 Introduction

Soil serves as the cradle of crop growth, and its health status greatly influences crop growth and development. From a long-term perspective, healthy and well-ordered soil directly determines the sustainable development of agriculture, ecological security, and even human well-being (Kibblewhite et al., 2008). As a vital component of terrestrial ecosystems, soil is not a static physical medium but a dynamic microenvironment teeming with life. Within this microenvironment, soil physical properties—such as aggregate stability (Xiao et al., 2022), porosity (Zhang et al., 2021), soil nutrients (Xi et al., 2021), as well as microbial community structure (Bardgett and van der Putten, 2014) and other factors are all subject to change due to external environmental influences, thereby affecting soil quality. The microbial communities present throughout the soil profile appear to serve as the core“drivers of healthy soil, playing a critical role in soil nutrient cycling and plant nutrition (Kiprotich et al., 2025). Through their complex networks and metabolic activities, beneficial microbial communities in the soil convert inert nutrients into available forms and construct soil aggregates that support soil structure (Fierer, 2017), thereby tightly integrating the physical, chemical, and biological properties of the soil into a unified organic whole (Young and Crawford, 2004).

Intensive agricultural systems were developed to meet the food and energy demands resulting from global population growth (Olson et al., 2017). It has been widely demonstrated that long-term intensive farming, particularly the continuous year-round cultivation of a single crop, leads to a decline in soil microbial community diversity and a simplification of soil structure (Huang et al., 2023; Yao et al., 2006) and disrupts its ecological balance. This imbalance often manifests as a reduction in beneficial symbiotic and antagonistic bacteria, while soil-borne pathogens such as *Fusarium* and *Rhizoctonia* accumulate and proliferate (Yang et al., 2023; Liu et al., 2014), thereby weakening the effective supply of soil nutrients (Wang et al., 2024), and ultimately leading to the problem of continuous cropping. However, crop continuous cropping problems are not an irreversible outcome; they can be mitigated through modern, well-established agronomic practices. Among these, crop rotation is a green and efficient soil improvement measure. A large body of literature has shown that crop rotation can alleviate the series of adverse soil effects caused by monoculture through crop diversification, thereby restoring soil health. For example, Xu et al. (2025) compared changes in soil microbial composition and nutrient levels between cucumber monoculture and cucumber-pea rotation systems. They found that beneficial bacteria such as *Bacillus* spp. in cucumber soils under rotation increased significantly, while the abundance of harmful fungi such as *Fusarium* spp. decreased, and soil nutrients under rotation increased significantly. Han et al. (2024) introduced diversified crops such as corn and tomatoes into continuous cotton fields and found that under rotation, soil organic carbon and organic matter contents increased significantly, soil aggregate structure improved, and the fungal community structure shifted. Similarly, D'Acunto et al. (2018) reported that increasing the number of rotation crops in soybean fields from two to four significantly enhanced soil microbial metabolic diversity, which was strongly positively correlated with plant biomass input. Sorghum, as the world’s fifth-largest cereal crop, possesses multiple stress-tolerant characteristics, including drought, waterlogging, and salinity tolerance. In Guizhou Province, sorghum serves as a key raw material for the production of Chinese baijiu and its cultivation area ranks among the highest in China (Chen et al., 2025). Renhuai City in Zunyi, as the core production area for organic sorghum in Guizhou Province, has developed various “sorghum-plus” crop rotation systems through long-term industrial practice. Among these, sorghum-potato, sorghum-green pea shoots, and sorghum-common vetch are three representative models. In particular, the sorghum-green pea shoots rotation has established a stable “dual-contract” cultivation model in the region, achieving an organic integration of land use and soil conservation. Although previous studies have explored various sorghum cultivation models focused on the green development of the sorghum industry, systematic and in-depth research on the rotational effects of these models remains lacking. Understanding of the soil fertility-enhancing effects of sorghum following rotation is also relatively limited. In particular, it is unclear how sorghum cultivation influences the direction of succession in soil microbial community structure under diversified rotation systems, and how these changes further drive improvements in soil nutrient status—the underlying mechanisms remain unclear. Based on this, this study uses sorghum as the core crop and selects potatoes, pea shoots, and vetch as the three rotation treatments. This approach not only reflects the actual planting needs of Guizhou’s major sorghum-producing regions but also covers three typical rotational functional types: tuber crops, leguminous vegetables, and leguminous green manure. This approach helps systematically elucidate the differentiated regulatory mechanisms of different rotation patterns on soil microbial communities and nutrient cycling.

Based on the above research background, this study proposes the following hypotheses: (1) The sorghum–potato rotation, due to its highest biomass return, can effectively enhance soil organic matter and total nutrient content; (2) Legume crop rotations can significantly improve soil nitrogen availability through biological nitrogen fixation; (3) All three rotation patterns can improve soil quality by increasing organic matter input and reshaping microbial community structure, but the effects of different rotation crops exhibit functional differentiation. By systematically investigating the effects of diversified crop rotations on the structure of soil microbial communities and soil nutrients in sorghum, this study aims to provide a theoretical basis and practical guidance for optimizing sustainable sorghum cropping systems, ecologically managing continuous cropping problems, and fostering healthy soils.

## 2 Materials and methods

### 2.1 Overview of the experimental site and design

The experimental field is located at the Shatian sorghum experimental plot in Shanshui Village, Luban Subdistrict, Renhuai City, Zunyi City, Guizhou Province (E106°17’, N27°42’). The study area has a subtropical humid monsoon climate with an annual average precipitation of 800–1,000 mm. The experimental site is flat, with an elevation of 915 m, with typical yellow soil. Sorghum was continuously planted from 2020 to 2023, with one crop per year and fallow in autumn and winter.

This experiment was conducted from 2023 to 2025 using a single-factor randomized complete block design. The experimental treatments included 6-year continuous sorghum (SSS), 4-year continuous sorghum-potato-sorghum (SPS), 4-year continuous sorghum-vetch-sorghum (SVS), and 4-year continuous sorghum-green pea shoots-sorghum (SGS) cropping systems. Each treatment was replicated three times, resulting in a total of 12 plots, each measuring 49 m^2^ (7 m × 7 m). Plots were arranged in a randomized block design with a 1.0-meter buffer zone between plots. The sorghum variety tested was Hongyingzi, the potato variety was Xisen No. 6, the vetch variety was Arrow-tongue Pea, and the green pea shoots variety was Black-sprouted Pea Tips. The cropping sequence over the two-year period was as follows: winter crops were planted in winter 2023, followed by sorghum in spring 2024; the same winter crops were planted again in winter 2024–2025, followed by sorghum in spring 2025. Final sampling was conducted in August 2025 at sorghum maturity.

As this experiment was conducted in an organic sorghum production base, no chemical fertilizers or pesticides were applied throughout the study. Field management practices were consistent across all treatments. For sorghum, basal fertilizer was applied before sowing at 2,250 kg∙ha^−1^ of commercial organic fertilizer (organic matter≥45%), followed by topdressing at 6,000 kg∙ha^−1^ at the jointing stage. For all three winter crops, basal fertilizer was applied at 750 kg∙ha^−1^ before sowing, with no topdressing. No irrigation was applied throughout the experiment, as the study area relies entirely on natural rainfall (annual precipitation 800–1,000 mm). Weeds were controlled manually, and pests and diseases were managed according to local organic standard practices without the use of chemical pesticides.

### 2.2 Test parameters and analytical methods

#### 2.2.1 Soil sampling and parameter measurement

##### 2.2.1.1 Soil collection and preservation

The grain-filling stage is critical for sorghum reproductive growth, when nutrient demand peaks, root activity is highest, root exudation is maximal, and rhizosphere microbes are most active. Sampling at this stage best reflects rotation effects on soil microbes and nutrients. All treatments were sampled simultaneously, ensuring fair comparison. Single-time-point sampling at the reproductive stage is widely adopted in crop rotation microbiome studies (Enagbonma et al., 2026). During the grain-filling stage of the 2025 sorghum season (August 15), three sorghum plants were randomly selected from each subplot. Using a sterile sampling shovel, a soil-attached root system was excavated in a circular pattern with a diameter of 20 cm and a depth of 30 cm around the base of the sorghum stems. The root system was gently lifted and shaken to remove loosely attached soil. A 2-cm-wide sterile soft-bristle brush was then used to collect tightly adhering soil along the direction of root growth. Mixed rhizosphere soil samples were prepared for each plot. Using tweezers, debris such as dead branches, fallen leaves, small stones, and residual roots were removed from the mixed samples, which were then passed through a 2-mm mesh sieve. The samples were placed in self-sealing bags, labeled with sample numbers, and stored in dry ice for rapid freezing before being immediately transported to the laboratory. Subsequently, one portion was stored in a − 80 °C ultra-low temperature freezer, while another portion was sieved and air-dried for long-term storage.

##### 2.2.1.2 Determination of soil physicochemical properties and methods

Upon returning the soil samples to the laboratory, the moisture content was immediately measured at 36.3%. The air-dried soil samples were then analyzed for physicochemical properties using the following methods: pH was determined by preparing a soil-water mixture at a 1:2.5 ratio and measuring with a pH meter (Liu et al., 2022). Organic matter was determined using the chromic acid redox titration method (external heat source method) (Fernandes et al., 2015). Total nitrogen was determined using the Kjeldahl method; total phosphorus was determined using the sodium hydroxide fusion-molybdenum-antimony colorimetric method;total potassium was determined using the flame photometric method; alkali-hydrolyzable nitrogen was determined using the diffusion absorption method; available phosphorus was determined using the 0.5 mol/L NaHCO₃extraction–molybdenum-antimony colorimetric method; and available potassium was determined using the NH₄OAC–flame photometric method (Jiang et al., 2025).

##### 2.2.1.3 High-throughput sequencing and data analysis

Soil samples were retrieved from a − 80 °C ultra-low temperature freezer, and total microbial community DNA was extracted using the CretMagTM Power Soil DNA Kit. After DNA extraction, the DNA concentration was measured using a TBS-380 fluorometer, DNA purity was assessed using a NanoDrop 2000 spectrophotometer, and DNA integrity was evaluated by 1% agarose gel electrophoresis. The primers 341F (5’-CCTAYSSSRBGCASCAG-3′) and 806R (5’-GGACTACHVSSSTWTCTAAT-3′) was used to amplify the V3-V4 variable regions of the 16S rRNA gene; The ITS region was amplified by PCR using primers ITS1 F (5’-CTTGGTCATTTAGAGGAAGTAA-3′) and ITS2R (5’-GCTGCGTTCTTCATCGATGC-3′). PCR amplification was performed in a 50 μL reaction volume containing 20 ng of diluted genomic DNA as template. After amplification, the products were purified and quantified. A library was constructed using the NEXTflex™Rapid DNA-Seq Kit (Bioo Scientific, United States) and sequenced on the Illumina (PE250) platform by Guizhou Bailoni Testing Technology Co., Ltd. Subsequently, analysis software such as FLASH, QIIME, and UPARSE was used to analyze microbial composition, abundance, and differences.

### 2.3 Data processing and analysis

Soil physicochemical data were organized and statistically analyzed using Microsoft Excel and one-way analysis of variance (ANOVA) and principal component analysis (PCA) were performed using SPSS 19.0 software. For high-throughput sequencing data, raw reads were quality-filtered using Fastp (Chen et al., 2018). Paired-end reads were assembled using FLASH (Magoč and Salzberg, 2011). Operational taxonomic units (OTUs) were clustered at 97% similarity using UPARSE (Edgar, 2013), and taxonomic annotation was performed against the SILVA (v138) (Quast et al., 2013) and UNITE (Nilsson et al., 2019) databases. Alpha diversity and beta diversity (Bray-Curtis PCoA) were calculated using QIIME2 (Bolyen et al., 2019). Fungal functional prediction was performed using FUNGuild (Nguyen et al., 2016). All statistical analyses and visualizations were conducted using R software. All data were based on three biological replicates (*n* = 3) and are presented as mean ± standard deviation. Differences among treatments were assessed using one-way ANOVA followed by Tukey’s HSD test, with a significance level of *p* < 0.05.

## 3 Results and discussion

### 3.1 Effects of diversified crop rotation on the physicochemical properties of sorghum rhizosphere soil

As shown in Table 1, compared with continuous sorghum cropping (SSS), the nutrient levels in sorghum rhizosphere soil under the three rotation systems—SSS, SVS, and SPS—exhibited varying trends. In the SPS rotation system (sorghum followed by potato), except for pH, which was lower than that of CK, soil organic matter, total nitrogen, total phosphorus, total potassium, alkali-hydrolyzable nitrogen, available phosphorus, and available potassium were all significantly higher than those of SSS (*p < 0.05*). In the SVS and SGS systems, soil total nitrogen, total phosphorus, alkali-hydrolyzable nitrogen, available phosphorus, and available potassium were all significantly higher than in SSS (*p* < 0.05), while soil pH and organic matter in these two rotation systems were significantly lower than in SSS (*p* < 0.05). Regarding total potassium, SGS was significantly higher than SSS, but there was no significant difference between SVS and SSS. Regarding available potassium, SGS was significantly higher than SSS, while SVS was significantly lower than SSS (*p* < 0.05). Among the three sorghum rotation systems, the soil physicochemical properties of SSS were higher than those of the SVS rotation system, while available potassium and alkali-hydrolyzable nitrogen in SGS were significantly increased compared to the other two rotation systems (*p* < 0.05). Overall, soil fertility under sorghum was significantly influenced by the rotation patterns, with SPS showing a greater improvement effect than the other two systems.

**Table 1:** Physicochemical properties of sorghum rhizosphere soils under different cropping systems.

Treatment	SSS	SPS	SVS	SGS
pH	7.50 ± 0.04a	7.16 ± 0.01b	6.63 ± 0.01c	6.24 ± 0.03d
Organic matter (g·kg^−1^)	47.25 ± 0.58b	53.29 ± 0.29a	45.48 ± 1.14c	42.33 ± 0.12d
Total nitrogen (g·kg^−1^)	2.77 ± 0.04d	3.37 ± 0.01a	3.20 ± 0.05b	3.08 ± 0.02c
Total phosphorus (g·kg^−1^)	1.08 ± 0.03b	1.48 ± 0.04 a	1.38 ± 0.04a	1.45 ± 0.05a
Total potassium (g·kg^−1^)	25.21 ± 0.30b	26.13 ± 0.63ab	25.37 ± 0.48b	26.80 ± 0.57a
Available nitrogen (mg·kg^−1^)	148.68 ± 0.68d	202.48 ± 0.39b	194.89 ± 0.66c	209.45 ± 1.65a
Available phosphorus (mg·kg^−1^)	75.25 ± 1.09d	97.67 ± 1.51a	80.27 ± 0.69c	85.03 ± 0.75b
Available potassium (mg·kg^−1^)	282.95 ± 1.37b	340.72 ± 3.41a	204.02 ± 2.40d	236.36 ± 1.08c

In the table, different lowercase letters indicate significant differences between treatments within the same soil layer (*p* < 0.05).

### 3.2 Effects of diversified crop rotation on the root-zone microbial Community of sorghum

#### 3.2.1 Petal-plot analysis of soil microbial OTU abundance

To investigate the distribution characteristics of shared and unique OTUs in the sorghum rhizosphere microbial community under different rotation systems, high-throughput sequencing analysis was performed on soil samples from different sorghum rotation treatments, and petal plots were constructed for bacterial and fungal communities based on OTU data. As shown in Figure 1, the number of shared bacterial OTUs across the four cropping systems was 1,812, while that of shared fungal OTUs was 363, indicating that the number of shared bacterial OTUs was significantly greater than that of fungi. Regarding bacterial OTUs, the number of unique OTUs in the SPS, SVS, and SGS treatments was significantly higher than that in the SSS (*p* < 0.05). Among these, the SVS treatment had 607 unique bacterial OTUs, which was significantly higher thanthose in the other rotation patterns. Regarding fungal diversity, the SVS treatment had 369 fungal-specific OTUs, which was significantly higher than in the other treatments (*p* < 0.05). These results suggest that the SVS treatment exhibited greater unique OTU richness in both bacterial and fungal communities compared with the other rotation systems, implying a potentially stronger response to crop rotation practices.

**Figure 1:**
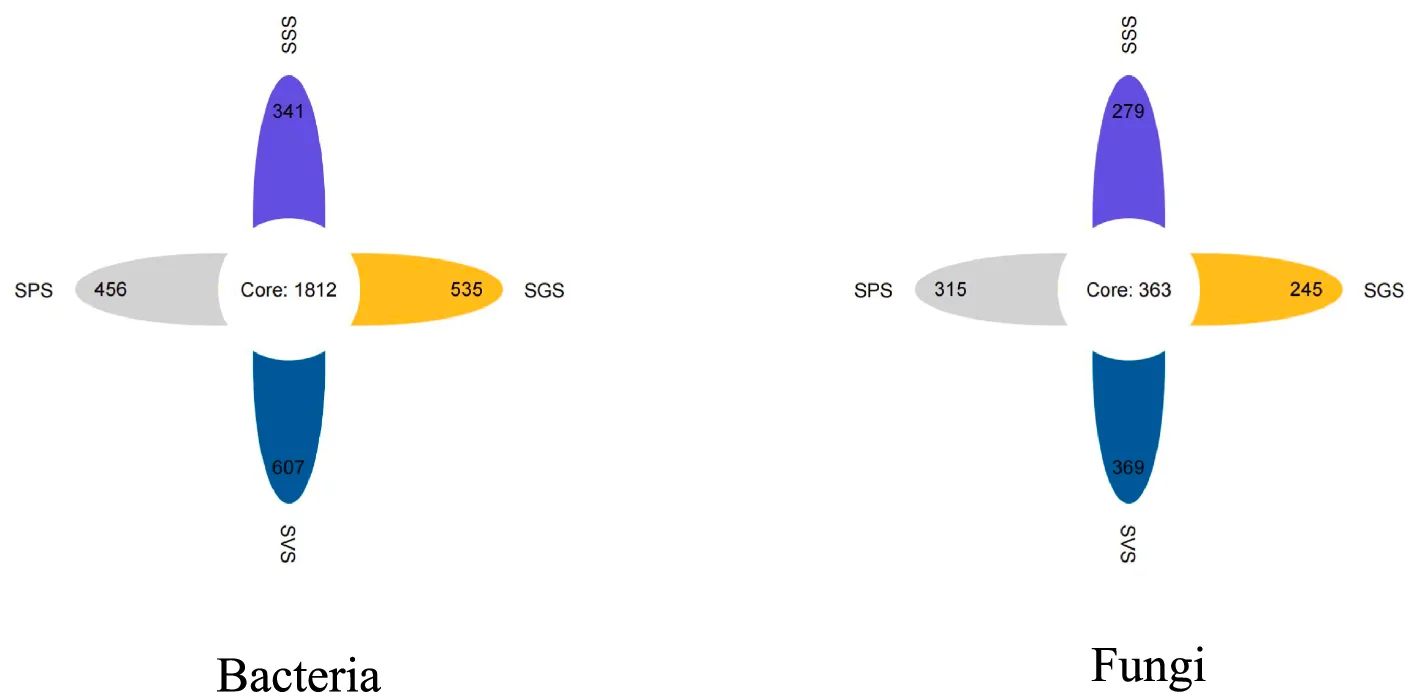
Petal plots showing the numbers of shared and unique OTUs of soil bacteria and fungi under different rotation patterns. Central numbers indicate OTUs shared among all four treatments. Numbers on the petals indicate unique OTUs in each treatment. OTUs were clustered using the UPARSE algorithm at 97% similarity threshold. Data were derived from three biological replicates per treatment (*n* = 3) and are presented as mean values.

#### 3.2.2 Analysis of alpha diversity in soil microbial communities

Alpha diversity analysis was conducted on the soil microbial communities in the rhizosphere of sorghum under different crop rotation treatments, with the results shown in Figure 2. The Good’s coverage for all treatments was greater than 0.99, indicating sufficient sequencing depth and reliable data. For soil bacterial alpha diversity (Figures 2A), in terms of species richness, both the Chao1 and Richness, and ACE indices all showed a pattern of SVS > SGS > SPS > SSS, with significant differences observed between treatments (*p* < 0.01). This suggests that crop rotation practices increased bacterial species richness compared with continuous cropping. For species diversity, the bacterial Shannon, Simpson, and Pielou indices under the SVS treatment were significantly higher than those in other treatments, followed by SSS, while bacterial diversity in the SPS and SGS treatments showed a decreasing trend. Notably, although SPS and SGS exhibited higher species richness than SSS, their diversity indices (Shannon, Simpson) were lower, suggesting reduced community evenness in these two rotation systems. For soil fungal alpha diversity (Figures 2B), in terms of species richness, the Chao1, Richness, and ACE indices all showed a pattern of SVS > SPS > SSS > SGS, with SGS exhibiting a highly significant downward trend (*p* < 0.01). In terms of species diversity, SPS had the highest relevant indices, while SVS and SGS had the lowest. Overall, SGS exhibited the lowest fungal richness and diversity among all treatments.

**Figure 2:**
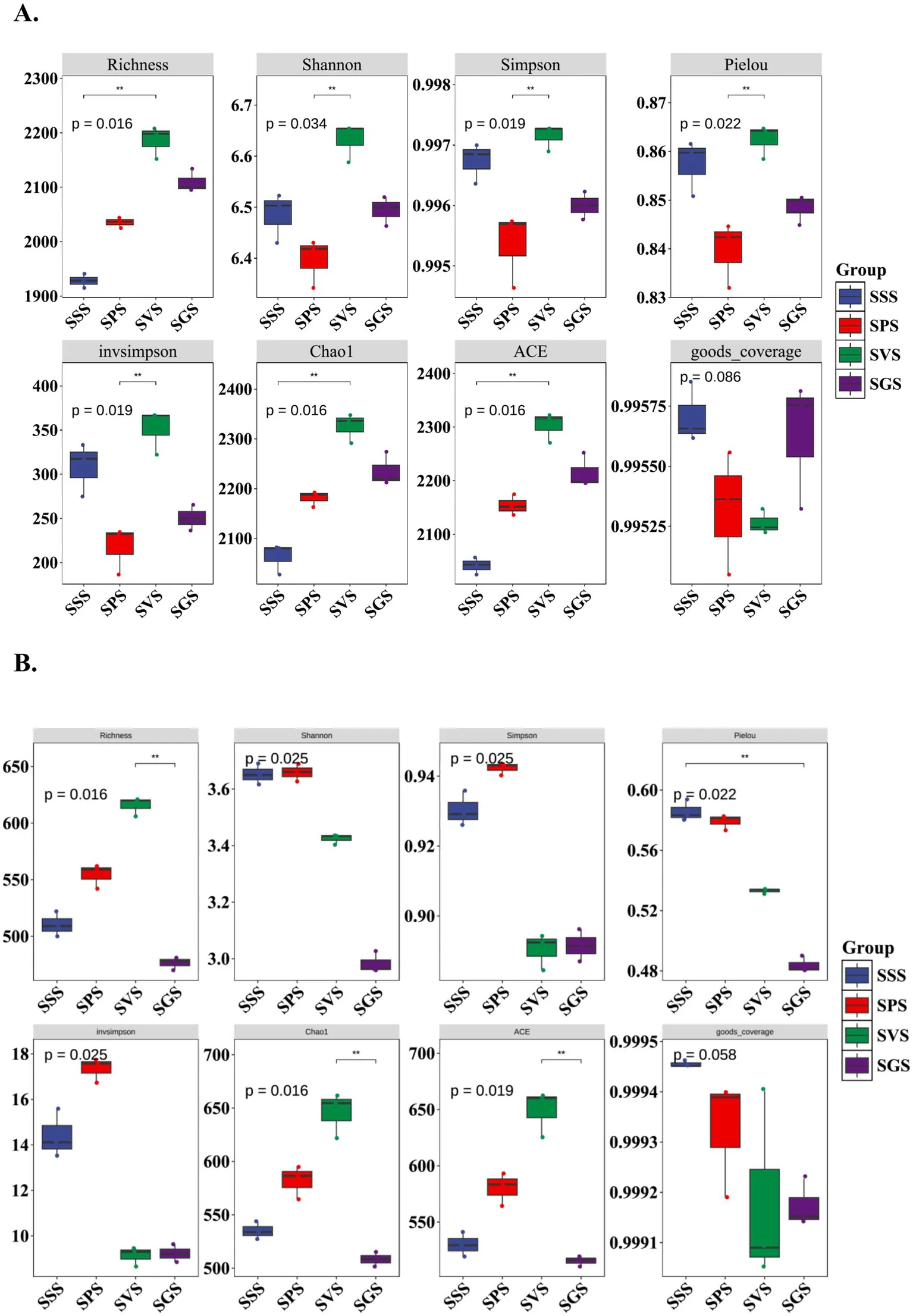
Alpha diversity of soil microbes in sorghum under different crop rotation systems. **(A)** Alpha diversity of bacteria; **(B)** Alpha diversity of fungi. **Indicates a highly significant difference (*p* < 0.01).

#### 3.2.3 Beta diversity analysis of soil microbial communities

Beta diversity analysis was conducted on the rhizosphere soil microbial communities of sorghum under different cropping systems. PCoA analysis based on Bray-Curtis distance (Figure 3) showed that for soil bacteria (Figure 3A), the first two axes explained 54.49 and 26.54% of the total variation, respectively, with a cumulative explanatory power of 81.03%. Samples from the SSS treatment were concentrated in the negative axis regions of PCoA1 and PCoA2, while samples from the SVS and SGS treatments were distributed in the positive axis region of PCoA1, and SPS samples were distributed along the positive axis of PCoA2. All three rotation treatments were completely separated from the SSS treatment. Adonis analysis indicated significant differences in soil bacterial community composition among treatments (*p* = 0.002). For soil fungi (Figure 3B), the cumulative explanatory power of the first two axes was 76.47%. Samples from the SSS treatment were located in the positive axis region of PCoA1, while SPS, SVS, and SGS samples were clearly separated from SSS along the PCoA2 axis. Adonis analysis indicated significant differences in soil fungal community composition among treatments (*p* = 0.001). However, the exceptionally high *R*^2^ value (e.g., 0.9979) is likely an artifact of overfitting due to the small sample size (*n* = 3) and should therefore be interpreted with caution. Overall, these results suggest that crop rotation tends to alter the overall structure of the soil microbial community in the sorghum rhizosphere, but the effect size should be considered exploratory evidence and requires validation with larger sample sizes.

**Figure 3:**
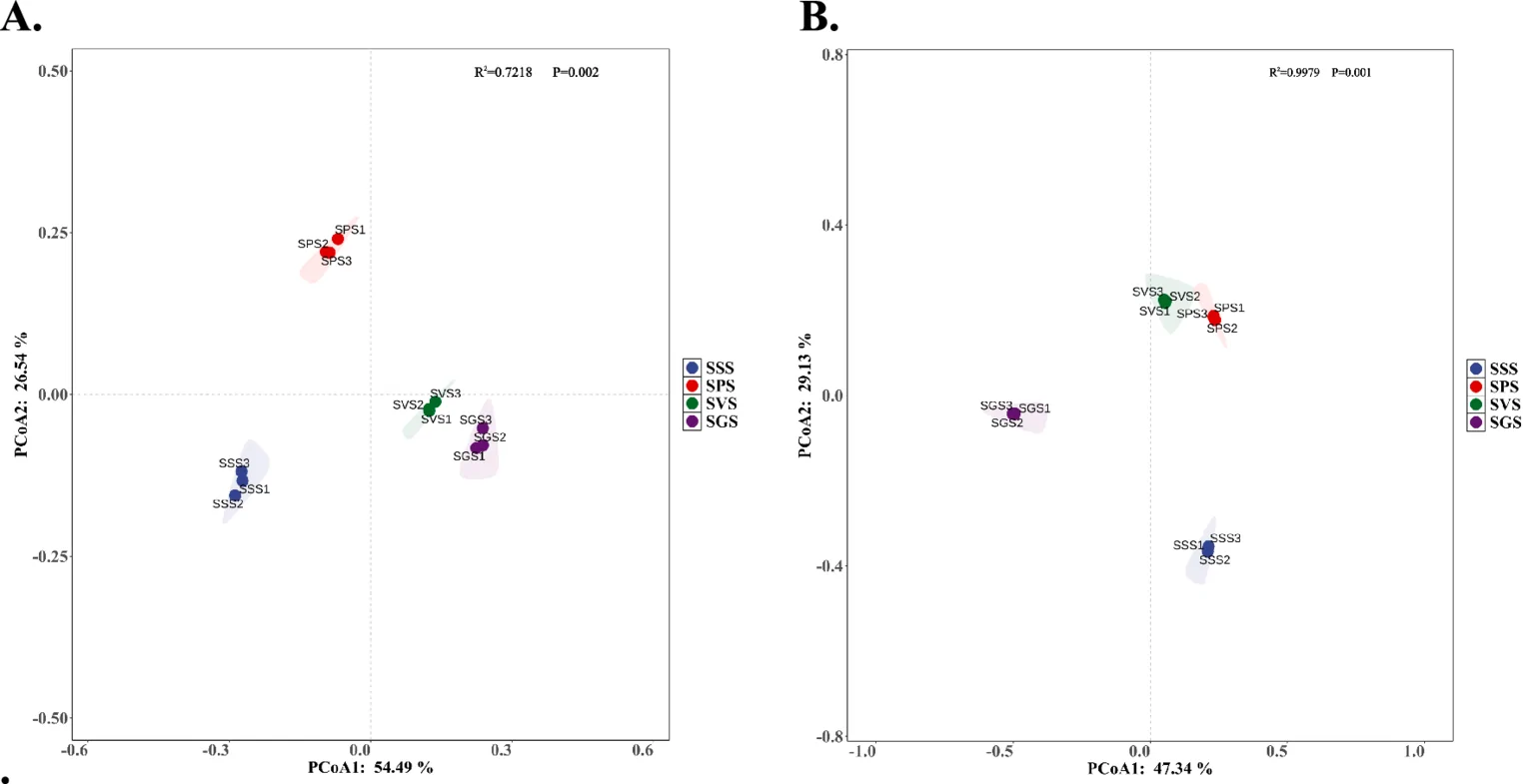
PCoA analysis of rhizosphere soil bacterial **(A)** and fungal **(B)** communities of sorghum under different planting patterns.

#### 3.2.4 Analysis of soil bacterial structure

As shown in Figure 4A, the top five dominant bacterial phyla in the rhizosphere soil of sorghum under different planting treatments were Proteobacteria, Actinobacteriota, Acidobacteriota, Gemmatimonadota, and Chloroflexi. A comparison of the relative abundances of dominant phyla across different treatments revealed that the relative abundance of Proteobacteria was 25.39% in SSS, increasing to 27.78%, 21.73%, and 28.05% in SPS, SVS, and SGS, respectively; the Actinobacteriota phylum had the highest relative abundance in SPS (27.72%) and the lowest in SSS (12.83%); The relative abundance of the Acidobacteriota phylum was highest in SSS (24.18%) and lowest in SPS (14.15%); The relative abundances of the Gemmatimonadota and Chloroflexi phyla ranged from 6.27 to 8.80% and 5.89 to 8.29%, respectively, across the different planting treatments.

**Figure 4:**
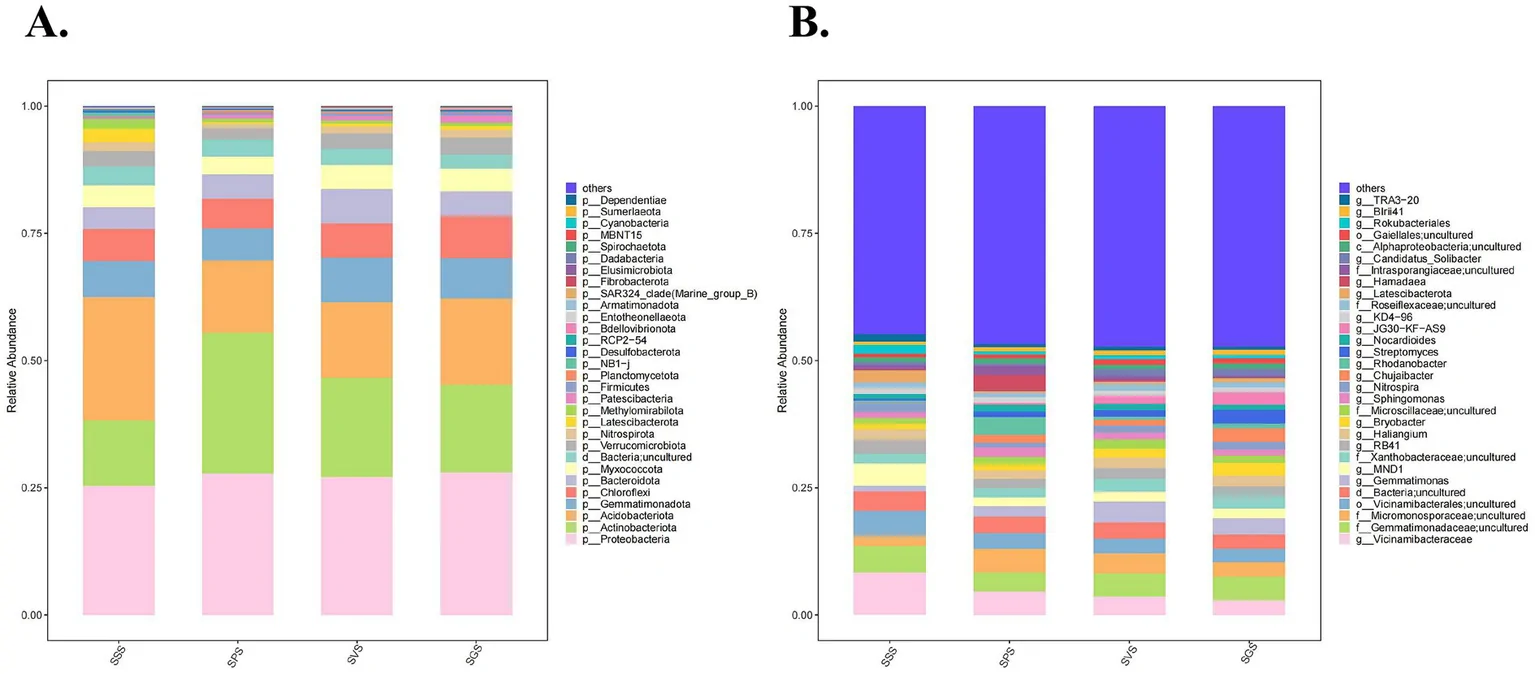
Bacterial community structure at the phylum level **(A)** and family level **(B)** in the rhizosphere soil of sorghum under different planting patterns.

As shown in Figure 4B, the dominant taxa at the family level (including the order-level taxon *Vicinamibacterales*) across different cropping systems included *Vicinamibacteraceae*, *Gemmatimonadaceae*, *Micromonosporaceae*, *Vicinamibacterales*, and unclassified Bacteria, with relative abundances ranging from 2.89–8.30%, 3.68–5.24%, 2.01–4.69%, 2.65–4.95% and 2.77–3.74%, respectively. Compared with SSS, the relative abundance of the *Micromonosporaceae* family increased by 0.96–2.68 and 0.92–2.06 percentage points in the three crop rotation treatments, respectively. The relative abundances of the remaining four dominant genera were highest in SSS, exceeding those in the three crop rotation treatments by 3.65–4.65, 0.68–1.56, 1.96–2.30, and 0.39–0.97 percentage points, respectively.

#### 3.2.5 Analysis of soil fungal structure

As shown in Figure 5A, the five dominant fungal phyla across the four cropping systems were Ascomycota, Basidiomycota, Mortierellomycota, unclassified Fungi, and Glomeromycota. Among these, Ascomycota had the highest relative abundance in SPS (90.40%) and the lowest in SGS (63.03%), with no significant difference between SVS and SSS; Basidiomycota had the highest relative abundance in SGS (33.09%), which was significantly higher than in the other three cropping systems, with no significant differences among SPS, SVS, and SSS; the Mortierellomycota and Unclassified Fungi had the highest relative abundances in SSS(8.26 and 3.97%, respectively), and the lowest in SGS (1.77 and 0.97%, respectively); the Glomeromycota also had the highest relative abundance in SSS (3.04%), while its relative abundance was extremely low in the three rotation treatments.

**Figure 5:**
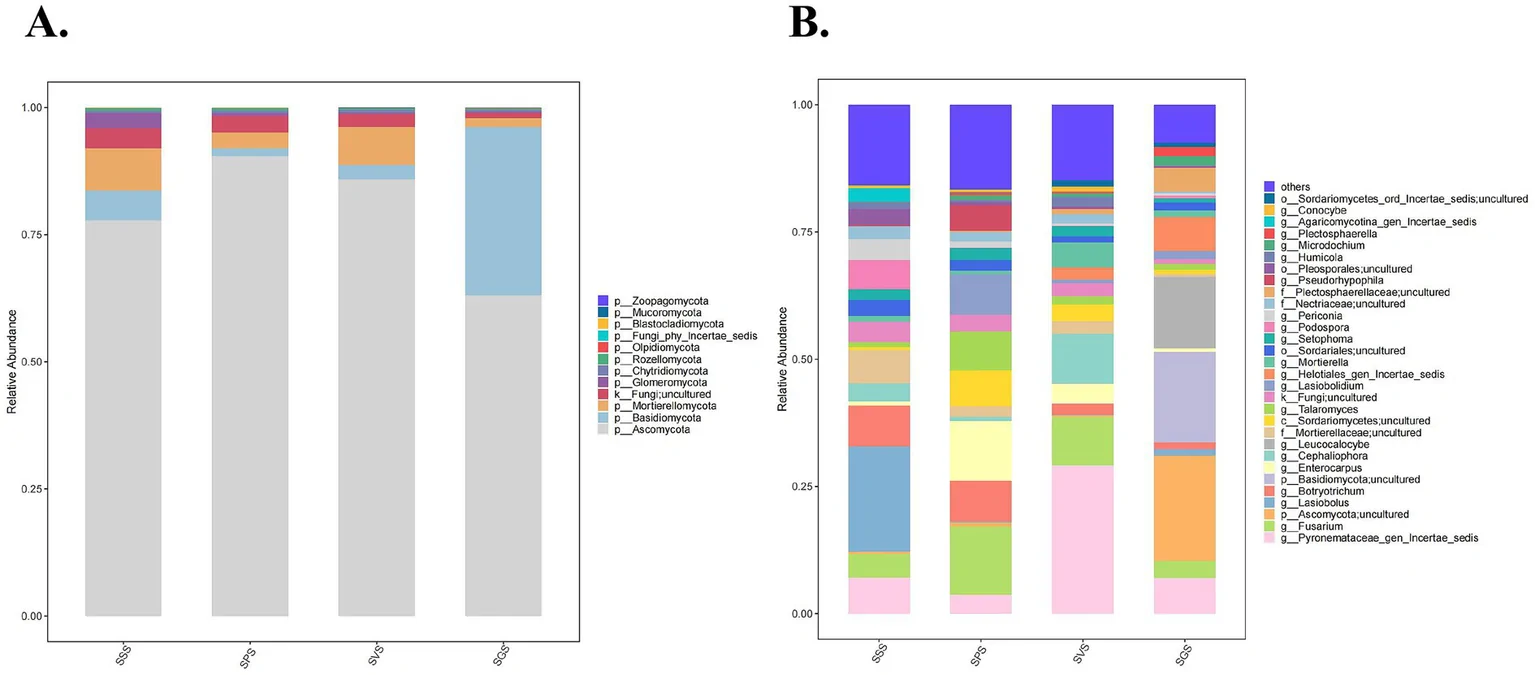
Fungal community composition in the sorghum rhizosphere soil under different cropping systems at the phylum level **(A)** and the dominant taxonomic groups **(B)**.

As shown in Figure 5B, the five dominant fungal taxa across the four cropping systems included the family *Pyronemataceae*, the genera *Fusarium*, *Lasiobolus*, and *Botryotrichum*, as well as uncultured Ascomycota. *Pyronemataceae* had the highest relative abundance in the SVS treatment (29.12%), which was 22.09%, 25.35%, and 22.10% higher than in the SSS, SPS, and SGS treatments, respectively; *Fusarium* had the highest relative abundance in the SPS treatment (13.32%) and the lowest in the SGS treatment (3.29%); The relative abundance of uncultured Ascomycota was highest in SGS (20.79%) and extremely low in the other three treatments; the genus *Lasiobolus* had the highest relative abundance in SSS (20.67%) and extremely low relative abundances in the three crop rotation treatments; the genus *Botryotrichum* had relative abundances ranging from 1.29 to 8.04% across the four treatments.

### 3.3 Correlation between soil nutrients and microbial community composition and diversity

To investigate the relationship between dominant microbial communities in soil and soil nutrients, we analyzed and compared the correlations between bacterial and fungal phyla and soil physicochemical parameters in the SSS and the crop rotation groups (SPS, SVS, and SGS). The results showed that the relative abundance of the Acidobacteriota was significantly negatively correlated with TN; the Gemmatimonadota was extremely significantly negatively correlated with AK; and the relative abundance of the Proteobacteria was significantly positively correlated with TP and extremely significantly positively correlated with AN (Figure 6A). The relative abundance of the Glomeromycota was significantly negatively correlated with both AN and TP; the relative abundance of unclassified Fungi was significantly positively correlated with pH (Figure 6B). The Mantel test was further employed to investigate the correlations between soil physicochemical factors and bacterial and fungal community structure. The results showed that both bacterial and fungal communities were significantly correlated with pH, TN, TP, TK, and AK (*p* < 0.05), with AK showing the strongest correlation, followed by pH and TN; whereas the correlations between SOM, AN, and AP and the microbial communities were not significant (*p* ≥ 0.05; Figures 6C,D). The results indicate that both continuous cropping and crop rotation systems exhibit significant correlations between dominant soil bacterial and fungal communities and the physicochemical properties of soil—namely, pH, SOM, TP, TN, AN, AP, and AK. Among these, pH, TN, TP, TK, and AK are key environmental factors driving changes in microbial community structure, with AK showing the most prominent correlation.

**Figure 6:**
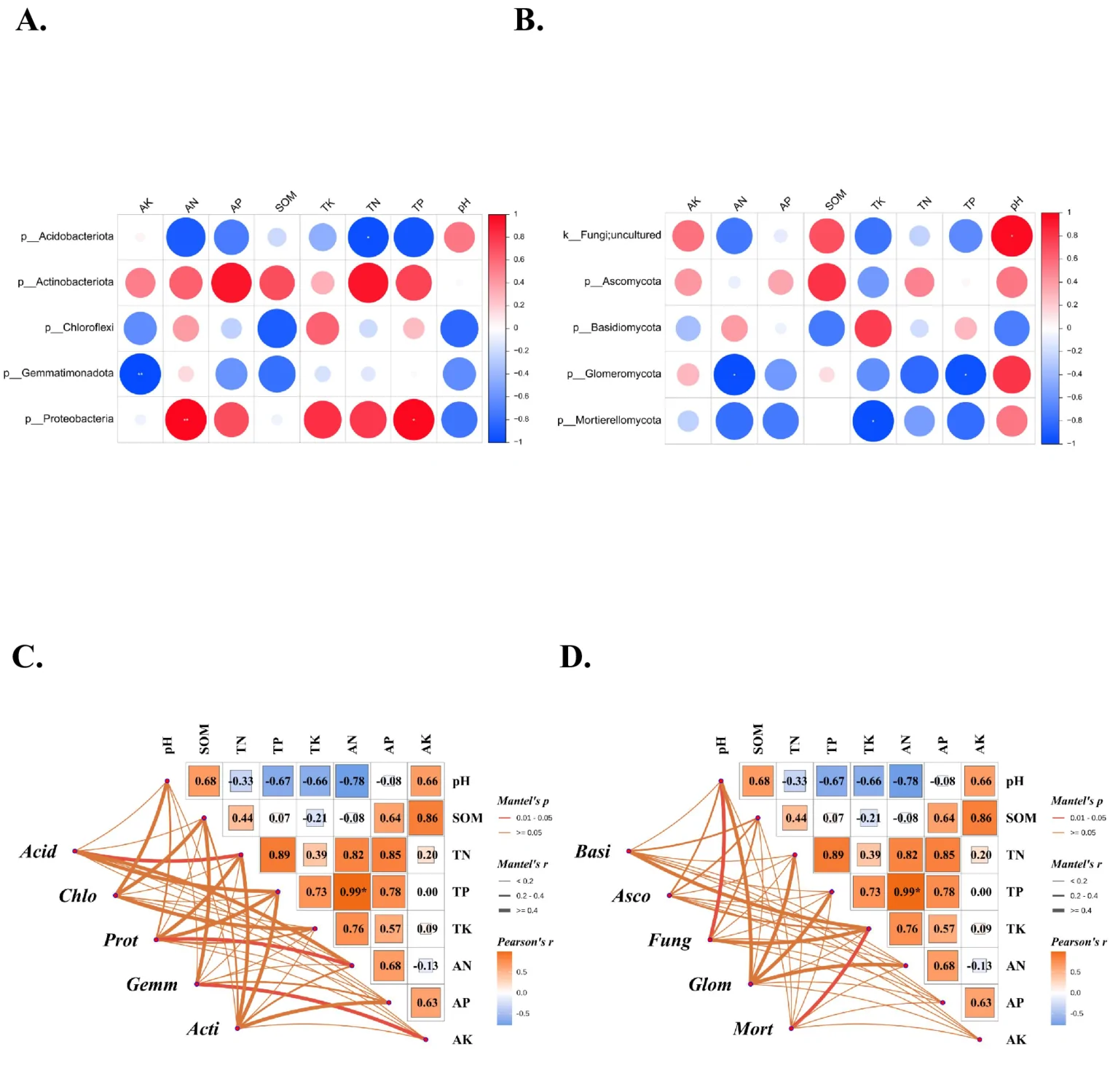
Correlation analysis between microbiota and physicochemical properties. **(A,B)** Correlations between relative abundance of soil bacteria and soil fungi at phylum level. **(C,D)** Mantel tests showing the relationships between soil bacterial and fungal community structures and soil properties. Bacterial phyla are abbreviated as follows: Acid, Acidobacteriota; Acti, Actinobacteriota; Chlo, Chloroflexi; Gemm, Gemmatimonadota; Prot, Proteobacteria. Fungal phyla are abbreviated as follows: Asco, Ascomycota; Basi, Basidiomycota; Fung, Fungi_unclassified; Glom, Glomeromycota; Mort, Mortierellomycota. AK, Available potassium; AN, Available nitrogen; AP, Available phosphorus; INV, Invertin; SOM, Organic matter; TK, Total potassium; TN, Total nitrogen; TP, Total phosphorus; *indicates a significant correlation at the *p* < 0.05 level; **indicates a significant correlation at the *p* < 0.01 level. The same applies below.

### 3.4 Microbial community functional analysis

FAPROTAX and FUNGuild were used to predict the functional composition of bacterial and fungal communities to elucidate the potential ecological roles of microorganisms in continuous cropping and rotation systems. The relative abundances of dominant functional groups revealed functional differences in soil bacteria and fungi between continuous cropping and crop rotation systems. As shown in Figure 7A, the dominant functional groups in the bacterial community (predicted by FAPROTAX) were chemoheterotrophy (36.97–38.21%), chemooxidotrophy (31.19–37.12%), predatory or exoparasitic (3.12–8.80%), nitrate reduction (1.45–6.98%), aromatic compound degradation (2.76–3.63%), nitrogen fixation (1.09–2.82%), and cellulolysis (0.52–4.26%). Compared with SSS, the relative abundances of chemoheterotrophy and aerobic chemoheterotrophy functional groups were significantly increased under all three rotation treatments; the nitrate-reducing functional group had the highest relative abundance in SPS (6.98%); the relative abundance of the nitrogen fixation functional group was highest in SVS (2.82%), followed by SGS; the relative abundance of the cellulolysis functional group was highest in SGS (4.26%). Among the dominant functional groups in the fungal community (predicted by FUNGuild; Figure 7B), the relative abundance of saprotrophic fungi was highest in the SVS treatment (9.84%, *p < 0.05*); endophytic fungi and lichen parasites had the highest relative abundance in SPS (13.33%, *p* < 0.05); the fungal functional group unique to the SGS treatment was ectomycorrhizal fungi and fungal parasites (14.08%, *p* < 0.05); and the relative abundance of plant pathogens in SSS was higher than in the other three rotation treatments (6.09%, *p* < 0.05). These results indicate that, regarding bacteria, crop rotation activated the metabolic capacity of soil microorganisms and enhanced functional groups associated with carbon and nitrogen cycling; regarding fungi, crop rotation effectively reduced the abundance of soil pathogens and enriched various beneficial fungal functional groups, such as saprophytic fungi and ectomycorrhizal fungi.

**Figure 7:**
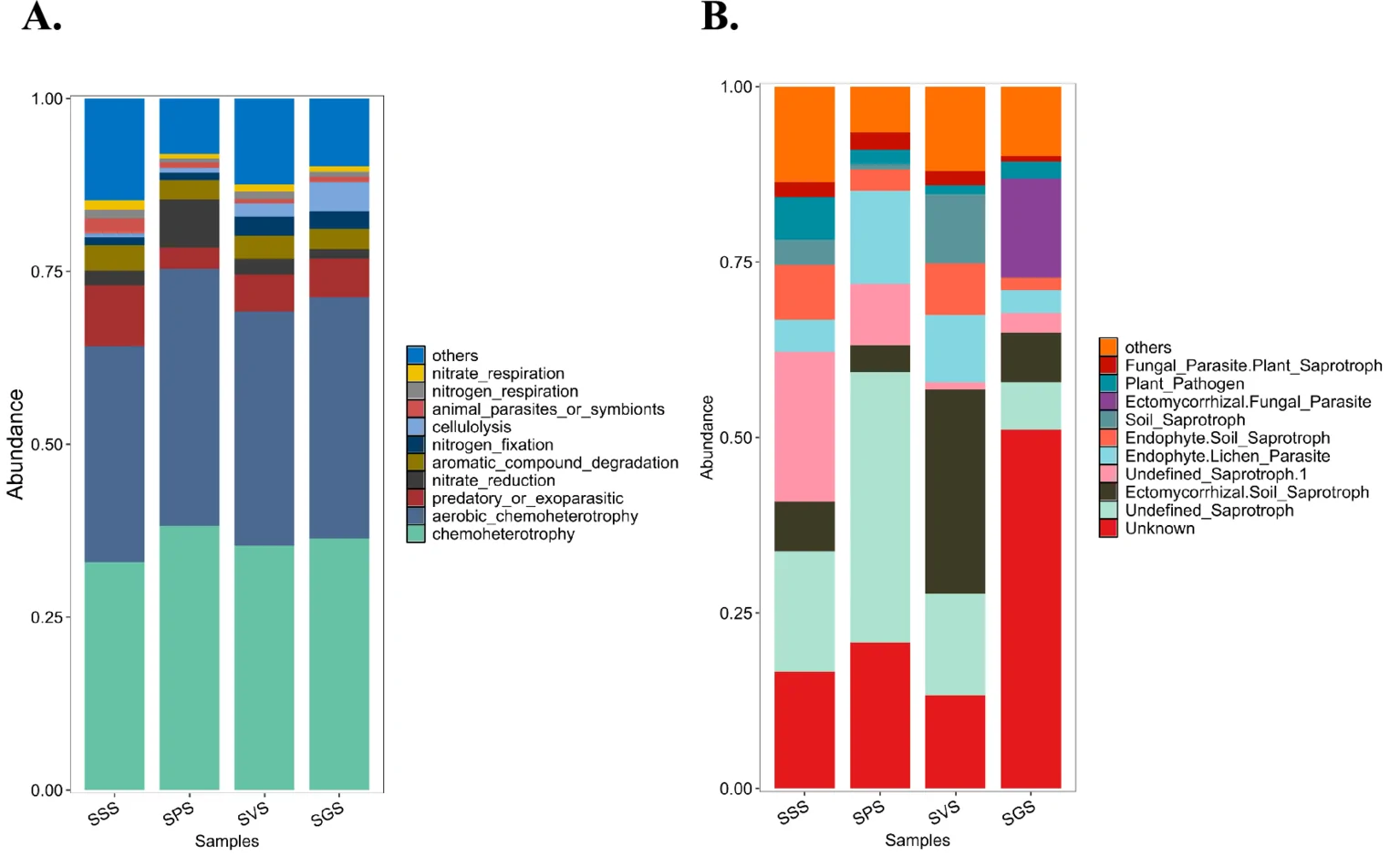
Functional composition of rhizosphere bacterial **(A)** and fungal **(B)** communities under different planting patterns.

### 3.5 Microbial co-occurrence network analysis

To further compare the underlying association patterns of microbial communities under continuous cropping and crop rotation conditions, a combined bacterial–fungal co-occurrence network was constructed based on Pearson correlation analysis (|r| > 0.6, *p* < 0.01; Figure 8). The SSS network comprised 400 nodes and 917 edges, of which 455 were positively correlated and 462 were negatively correlated; the complete crop rotation network comprised 402 nodes and 1,046 edges, of which 696 were positively correlated and 350 were negatively correlated. The proportion of positively correlated edges increased from 49.6% in the SSS to 66.5% in the rotation network, while the proportion of negatively correlated edges decreased from 50.4 to 33.5%. For ease of visualization, only the positively correlated subnetworks are shown, with the SSS network comprising 330 nodes and 455 edges, and the rotation network comprising 357 nodes and 696 edges.

**Figure 8:**
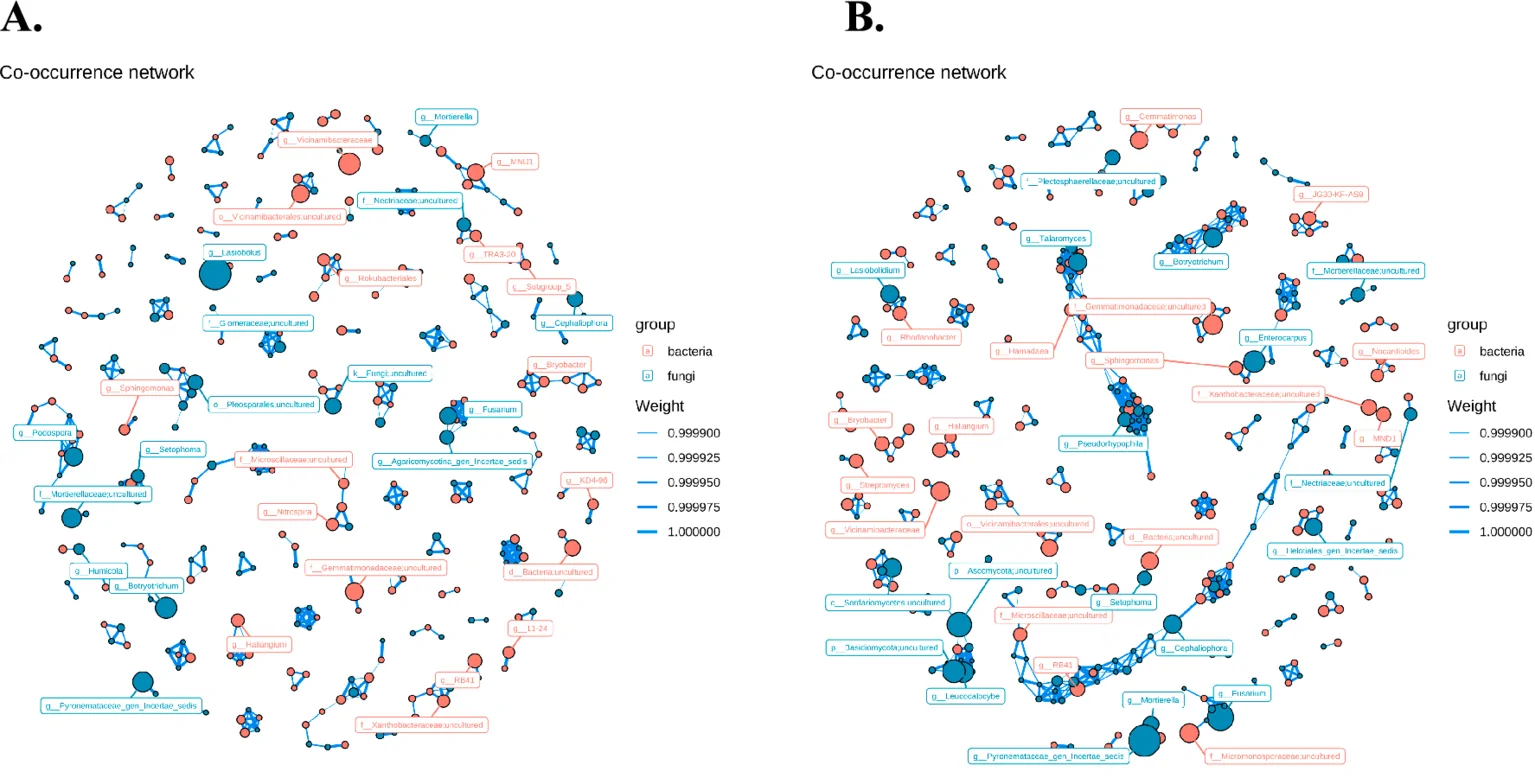
Bacterial–fungal co-occurrence networks under the SSS **(A)** and rotation treatment **(B)** in sorghum. Networks were constructed based on Pearson correlation analysis (|r| > 0.6, *p < 0.01*). Red nodes represent bacteria; cyan nodes represent fungi. Node size is proportional to relative abundance. Edges indicate significant correlations. Only positive correlations are shown for clarity. SSS, continuous cropping; Rotation: combined samples of SSS, SVS, and SPS treatments.

Tpological analysis of the complete networks (Table 1) showed that the average degree for the SSS and rotation networks was 4.59 and 5.20, respectively; network density was 0.0115 and 0.0130, respectively; average clustering coefficients were 0.677 and 0.703, respectively; and modularity was 0.738 and 0.794, respectively. The largest connected component in the rotation network comprised 49 nodes, a number higher than that in the SSS network (29 nodes). These results indicate that the rotation network exhibits higher overall connectivity, local clustering, and modularity under the current network construction conditions. Node centrality analysis further revealed that certain microbial groups in the rotation network exhibited high topological importance. Among them, *Pseudorhypophila* had a high degree (16), indicating that it was a highly connected candidate hub group; *Cephaliophora* exhibited high betweenness centrality (567), suggesting it may play a potential bridging role between different network substructures. Thus, these groups should be regarded as candidate topologically important groups rather than ecologically key species in the strict sense.

Tpological analysis of the complete networks (Table 2) showed that the average degree for the SSS and rotation networks was 4.59 and 5.20, respectively; network density was 0.0115 and 0.0130, respectively; average clustering coefficients were 0.677 and 0.703, respectively; and modularity was 0.738 and 0.794, respectively. The largest connected component in the rotation network comprised 49 nodes, a number higher than that in the SSS network (29 nodes). These results indicate that the rotation network exhibits higher overall connectivity, local clustering, and modularity under the current network construction conditions. Node centrality analysis further revealed that certain microbial groups in the rotation network exhibited high topological importance. Among them, *Pseudorhypophila* had a high degree (16), indicating that it was a highly connected candidate hub group; *Cephaliophora* exhibited high betweenness centrality (567), suggesting it may play a potential bridging role between different network substructures. Thus, these groups should be regarded as candidate topologically important groups rather than ecologically key species in the strict sense.

**Table 2:** Topological properties of bacterial–fungal co-occurrence networks.

Parameter	SSS	Rotation
Nodes	400	402
Edges	917	1,046
Average degree	4.59	5.20
Modularity	0.738	0.794
Largest connected component	29	49

Topological parameters were calculated from the node and edge files exported by the plotting platform. Modularity was calculated using the absolute value of the Pearson correlation coefficient as edge weight. Node centrality metrics were recalculated to identify candidate taxa with important topological significance.

## 4 Discussion

### 4.1 Analysis of the effects of different treatments on the physicochemical properties of sorghum rhizosphere soil

Soil nutrients, as the material foundation of healthy soil, are the core elements that sustain soil fertility (Nawaz et al., 2026). They serve not only as the cradle of plant growth but also as the energy source that drives soil microbial metabolic activities and maintains the normal functioning of the entire soil ecosystem (Dilly, 2005). Soil physicochemical properties, as fundamental indicators of soil fertility, directly influence crop growth and the structural composition of soil microorganisms. This study, which examined sorghum rotation with different crops, found that compared with the control group (SSS), the three rotation treatments (SPS, SVS, and SGS) all altered the physicochemical properties of the soil in the sorghum rhizosphere to varying degrees. This is consistent with previous research on crop rotation soils, such as that by Liu et al. (2026), who found that in a watermelon-field bean rotation system, the organic matter, total nitrogen, alkali-hydrolyzable nitrogen, and available phosphorus in watermelon fields under rotation, as well as the soil quality index, were significantly improved. Liu J. et al. (2025), who, through studies on Corydalis rotations with different crops, found that various physicochemical properties of Corydalis soil, including organic matter, were enhanced under rotation, while enzyme activity in the rotated soil was also significantly increased. These results collectively indicate that crop rotation between different crops can effectively regulate and improve soil nutrients. Notably, organic matter, total phosphorus, total nitrogen, total potassium, and available nutrients in the SPS were all comprehensively enhanced, suggesting that the sorghum-potato rotation system is most effective in improving soil nutrients. This may be attributed to the fact that potatoes are tuber crops, with plant biomass far exceeding that of green manure and peas. Consequently, they return a greater amount of organic residue to the soil after harvest, increasing organic matter input, attracting more beneficial soil microorganisms, and promoting the enhancement of soil nutrients (Xiao et al., 2022); the content of alkali-hydrolyzable nitrogen in SGS was significantly higher than in other treatments. This is because peas are leguminous crops, their root exudates recruit nitrogen-fixing bacteria to colonize the roots, forming root nodules that convert atmospheric N₂ into plant-available nitrogen sources (Tao et al., 2024). However, as the pea plants were harvested at the seedling stage for edible shoots, the extent of nitrogen fixation may be limited, and further investigation is needed to clarify the underlying mechanisms. Soil nitrogen and phosphorus content are key indicators of soil productivity and are also critical nutrients for sorghum plant growth (Hu et al., 2023). In this study, both soil alkali-hydrolyzable nitrogen and available phosphorus were higher under all rotation treatments than in SSS, indicating that rotating sorghum with other crops effectively enhances soil levels of readily available nutrients, thereby laying a nutritional foundation for sorghum growth.

It is worth noting that soil pH under all rotation treatments in this study was lower than in SSS, with the most significant decrease observed in the SGS treatment (from 7.50 to 6.24), followed by the SVS treatment. This may be related to the biological characteristics of green manure and peas, as both belong to the Fabaceae (legume) family; their root nodule nitrogen fixation releases H^+^, leading to localized soil acidification (Bolan et al., 1991). The localized acidification induced by biological nitrogen fixation (BNF) in legumes has been shown to significantly alter the composition and abundance of microbial communities in the adjacent rhizosphere. Xia et al. (2026) demonstrated through cross-regional meta-analysis and controlled experiments that soil pH is the strongest predictor of rhizobial community variation in the legume rhizosphere. They further showed that pH manipulation significantly alters the taxonomic composition of rhizobial communities, with acidic conditions favoring the enrichment of *Bradyrhizobium*, while alkaline conditions promote the colonization of *Rhizobium* and *Mesorhizobium*. Although the decrease in soil pH reflects a trend toward soil acidification to some extent, soil nutrient indicators under all rotation treatments in this study were generally superior to those of SSS, indicating that within the pH range of 6.24–7.16, the soil maintained good nutrient-supplying capacity. Furthermore, the available potassium levels in both the SVS and SGS treatments were significantly lower than those in SSS. This may be because green manure and peas are potassium-demanding crops that require large amounts of potassium for their own growth. Studies by Divito and Sadras (2014) and others have shown that potassium is a key element for enhancing biological nitrogen fixation efficiency in leguminous crops, and potassium deficiency can severely inhibit nitrogen fixation. These findings suggest that in sorghum-legume rotation systems, attention should be paid to trends in soil acidification and potassium deficiency. Where necessary, acid-neutralizing measures such as lime application should be implemented, and potassium application rates should be increased during the sorghum growing season.

### 4.2 Effects of different treatments on the taxonomic abundance of microbial communities

Microbial community diversity and structure are important biological indicators for evaluating soil health (Wooliver et al., 2025). Previous studies in numerous crop rotation experiments have confirmed that crop-diversified rotations can improve soil health by reshaping the soil microbial community. For example, Zhou et al. (2026) found in a rice rotation system that paddy field rotation influences the relationship between rice yield and ecosystem functions by reshaping the assembly processes and functional characteristics of the paddy soil microbial community. Liu G. Y. et al. (2025) confirmed through a four-year field experiment that a diversified wheat rotation system significantly increased the abundance and diversity of the soil microbial community and enhanced the complexity and stability of microbial networks. In this study, petal plot analysis showed that the number of bacterial OTUs in the crop rotation treatments was higher than in the continuous cropping treatment, which is consistent with previous findings that crop rotation can increase soil bacterial richness (Li et al., 2025). A meta-analysis based on 148 global studies confirmed that crop rotation significantly increases bacterial species richness. Crop rotation increases the heterogeneity of soil carbon sources and ecological niches through the input of diverse root exudates and crop residues, while simultaneously improving soil pH and nutrient availability, thereby supporting the colonization of a greater number of bacterial groups. Regarding fungi, the number of unique OTUs in the SPS and SVS treatments was higher than in the continuous cropping treatment, which may be related to the distinct residue quality of potato and common vetch. As a tuber crop, potato residues are rich in cellulose and hemicellulose, providing selective substrates for specific fungal groups (Li et al., 2023). Common vetch, as a leguminous green manure crop, is incorporated into the soil as whole plants, and its readily degradable organic matter and nitrogen-fixing activity improve soil carbon and nitrogen supply, promoting the diversification of fungal communities (Yu et al., 2022). The SVS treatment exhibited the highest number of unique OTUs for both bacteria and fungi. This may be attributed to the fact that the entire vetch plant was incorporated into the soil, introducing a large amount of readily degradable organic carbon and biologically fixed nitrogen, thereby providing bacteria and fungi with more abundant carbon and nitrogen sources and supporting the colonization and enrichment of more unique taxa. The Alpha diversity analysis in this study revealed that, compared with SSS, all three rotation treatments significantly increased the species richness of bacterial communities. This may be attributed to the fact that crop rotation introduces diverse root exudates and crop residues, providing soil microorganisms with more abundant carbon sources and nutrients. Moreover, the different root morphologies of various crops break the monopoly of a single crop on specific ecological niches, offering broader survival space for bacteria with different ecological strategies. Additionally, crop rotation improves soil physicochemical properties, creating a more suitable environment for the colonization and reproduction of more species. However, the effects of different rotation patterns on bacterial and fungal communities differed significantly. Specifically, soil bacterial richness and diversity were higher under the SVS treatment than under other treatments. This may be attributed to the abundance of readily available organic compounds (e.g., amino acids and organic acids) in the root exudates of leguminous green manure crops, which support the growth of a wider range of microbial taxa (Bobille et al., 2016). However, among leguminous crops, bacterial richness and diversity under the SGS treatment were lower than those under the SVS treatment. This may be because the peas in the SGS treatment are leguminous crops also used for vegetable production, the removal of their edible aboveground parts may alter the growth status of the plant roots (Dollete et al., 2025), thereby affecting the formation and release efficiency of root exudates (Thilakarathna and Raizada, 2019). Furthermore, since arrow-tongue vetch are incorporated into the soil as green manure with the entire plant plowed under, the amount of biomass returned to the soil is far greater than that of peas where only the stubble is left in the field. This introduces richer organic matter and nutrients into the soil, thereby influencing the differences in bacterial structure between the two. This difference is consistent with the findings of Lupwayi and Soon (2016), which compared the effects of seed-harvested peas and arrow-tongue vetch as green manure on soil microorganisms and found that the green manure treatments significantly outperformed the seed-harvested pea treatments in terms of increasing microbial biomass carbon and enzyme activity. Notably, bacterial richness under both SPS and SGS treatments was higher than that of SSS, yet bacterial diversity was lower than that of SSS. This result contradicts previous studies and may be attributed to the short duration of the crop rotation. Although SPS and SGS treatments introduced more new bacterial species, the abundance of these introduced species was low, leading to a decrease in the overall evenness of the community and consequently resulting in lower bacterial diversity under these two treatments compared with the control. Maciá-Vicente et al. (2024) found that short-term intercropping with cover crops did not increase microbial diversity or community differentiation. This further confirms that the reshaping of microbial communities through crop rotation is a process requiring long-term accumulation; therefore, these findings should be interpreted with caution.

Regarding the bacterial microbial composition, this study compared continuous sorghum cropping with crop rotation and found that all three rotation systems increased the relative abundances of the Proteobacteria and Actinobacteriota phyla in the soil, while decreasing the relative abundance of the Acidobacterotia phylum. Shi et al. (2026) demonstrated through a five-year field experiment that legume rotation enriched Actinobacteria and Proteobacteria in the potato rhizosphere to 38.31 and 28.40%, respectively, while reducing Acidobacteria to 10.03%, which is highly consistent with our findings. This may be attributed to the incorporation of diverse plant residues under rotation, which provided copiotrophic bacteria such as Proteobacteria and Actinobacteria with abundant carbon sources and available substrates, thereby promoting their proliferation (Yang et al., 2022). The Proteobacteria and Actinobacteriota phyla are widely distributed in nature and constitute important functional microbial communities in soil. Among them, Proteobacteria play a significant role in soil carbon, nitrogen, and sulfur cycles, promoting plant growth and improving the soil ecological environment (Khan et al., 2023), while the secondary metabolites secreted by Actinobacteriota can effectively enhance soil fertility and inhibit the proliferation of soil-borne pathogens (Salwan and Sharma, 2020). Shi et al. (2026) confirmed through metagenomic analysis that fava bean-potato rotation significantly enriched the Actinobacteriota and Proteobacteria phyla in the potato rhizosphere while reducing the Acidobacteriota phylum. This finding is consistent with the results of this study, indicating that the enrichment of beneficial soil bacteria can improve soil health and enhance ecological functions. Regarding fungal microorganisms, the Ascomycota phylum exhibited the highest relative abundance in SPS. Studies have shown that ascomycetes, as the primary decomposers in soil, secrete extracellular enzymes such as cellulases and lignin peroxidases, playing a key role in degrading refractory organic matter in plant residues, such as lignin and cutin (Shi et al., 2026; Osono et al., 2021). In SPS, potatoes, as a tuber crop, have high levels of cellulose and hemicellulose in their residues, providing a rich substrate for the growth of Ascomycota. This may be one of the reasons for the highest relative abundance of Ascomycota in this treatment. The Basidiomycota are one of the major components of the soil fungal community. They are generally considered important saprophytes in soil ecosystems and are capable of forming mycorrhizal symbiotic relationships with plant roots, playing a key role in soil nutrient cycling (Clasen et al., 2018; Boddy et al., 2008). Basidiomycetes were significantly enriched in the SGS treatment, which not only promoted the decomposition of recalcitrant organic matter in the soil and accelerated nutrient release but also enhanced the absorption of mineral nutrients through the formation of mycorrhizal networks with plant roots, thereby improving soil fertility and promoting the growth of the subsequent sorghum crop. Similarly, members of the family *Pyronemataceae*, known as typical saprotrophic fungi, also possess the ecological function of decomposing organic matter and driving nutrient cycling (Fischer et al., 2021). This family exhibited the highest relative abundance in the SVS treatment, indicating that different rotation patterns can recruit diverse beneficial fungal communities to perform equivalent ecological functions, thereby jointly promoting soil nutrient cycling and the maintenance of ecosystem functions. Notably, the pathogenic fungus *Fusarium* exhibited the highest relative abundance in the SPS rotation treatment and the lowest in the SGS treatment. This result indicates that different rotation crops exert significantly different regulatory effects on soil-borne pathogens; the significant reduction in *Fusarium* abundance in the SGS treatment is one of the key ecological advantages of pea rotation. While the SPS rotation, although capable of comprehensively improving soil nutrients, is less effective than other rotations in suppressing soil-borne diseases. This phenomenon may stem from the fact that potatoes are susceptible hosts for *Fusarium*. Studies have shown that *Fusarium acuminatum* is a soil-dwelling bacterium with a broad host range that can saprophytically colonize various plant residues, potatoes provide this pathogen with a suitable substrate for proliferation through their root exudates and plant residues (Song et al., 2026). Therefore, the disease-suppressing effect of SPS crop rotation is not consistently evident under all conditions, and its effectiveness may be constrained by numerous factors. For example, when the rotation period is short (only 2 years), the pathogen population accumulated in the soil may not yet have been effectively reduced (De Corato et al., 2020). Alternatively, the risk of disease may increase significantly when potato varieties lack sufficient resistance. This suggests that in practical production, if the sorghum-potato rotation system is adopted, it is recommended to combine it with auxiliary measures such as disease-resistant varieties, biological agents, or soil disinfection to effectively control the risk of *Fusarium* diseases. At the same time, long-term field trials should be conducted to further clarify the disease-suppressing effects of the SPS rotation system over different durations and under various soil conditions, as well as its underlying mechanisms of action.

### 4.3 Effects of different treatments on the functional profiles of microbial communities

Microbial functional communities are the fundamental drivers of soil nutrient cycling and the maintenance of soil microecological health; changes in these communities directly reflect the regulatory effects of crop rotation measures on soil ecological functions (Sahu et al., 2019). In this study, FAPROTAX and FUNGuild functional prediction analysis revealed that different crop rotation treatments significantly altered the composition of functional groups of soil bacteria and fungi, and different rotation patterns exhibited distinct differentiation characteristics at the functional level. Regarding bacterial functions, all three crop rotation treatments significantly increased the relative abundances of chemoheterotrophy and aerobic chemoheterotrophy functional groups. As chemoheterotrophy is the core metabolic pathway of soil heterotrophic microorganisms, its functional enhancement implies accelerated rates of soil organic matter decomposition and nutrient mineralization, which is conducive to rapid improvements in soil fertility. Studies by Liu et al. (2023) found that straw mulching in maize fields significantly increased the relative abundance of chemoheterotrophic and chemooxidotrophic bacteria in the soil. The enhancement of these functional groups directly drives soil carbon and nitrogen cycling processes and ultimately increases the content of active carbon and nitrogen in the soil. The results of this study indicate that crop rotation measures provide soil microorganisms with a richer source of carbon and energy by increasing the input of crop residues and root exudates, thereby comprehensively activating the basal metabolic capacity of soil microorganisms. The relative abundance of nitrate-reducing functional groups was highest in the SPS. Previous studies have found that the activity of nitrate-reducing functional groups directly influences the form and content of inorganic nitrogen in the soil, thereby regulating the efficiency of crop nitrogen uptake and utilization (Du et al., 2024), while nitrogen-fixing functional groups exhibited the highest relative abundance in SVS and SGS, indicating that different crop rotation patterns regulate soil nitrogen transformation processes by specifically enriching distinct nitrogen cycling functional groups.

Regarding fungal functions, One-way ANOVA followed by Tukey’s HSD test revealed significant differences in multiple fungal functional guilds among treatments (Figure 8B), indicating that all three rotation systems enhanced soil fertility and maintained soil health through the diversified enrichment of fungal functional guilds. Among these, saprophytic fungi had the highest relative abundance in the SVS treatment, promoting the rapid decomposition of green manure residues and the release of nutrients; while SPS enhances nutrient uptake and stress resistance in the subsequent sorghum crop through the enrichment of the endophytic-lichen parasitic composite functional group (Perkins et al., 2021). Studies have shown that endophytic and lichen-parasitic fungi, which are commonly classified within the phylum Ascomycota, possess both symbiotic and parasitic functions (Arnold et al., 2009; Tripathi and Joshi, 2019). This may also be one of the reasons why the Ascomycota phylum exhibits the highest relative abundance in SPS; whereas the ectomycorrhizal-mycopathogenic fungal functional group, unique to SGS, enhances plants ability to absorb soil nutrients by forming symbiotic relationships between ectomycorrhizal fungi and plant roots, while its mycopathogenic functional group exerts a direct biological control effect on soil-borne pathogens (Singh et al., 2024), which may also be one of the reasons for the lowest abundance of soil-borne pathogens such as *Fusarium* under the SGS treatment. Notably, the relative abundance of plant pathogens in the SSS treatment was significantly higher than in the three crop rotation treatments. This result confirms that crop rotation is an effective measure for reducing soil-borne diseases and alleviating crop continuous cropping stress. This finding is consistent with previous studies; for example, Xiong et al. (2017) found that crop rotation can suppress pathogen proliferation by reshaping the structure of the soil microbial network.

## 5 Conclusion

In this study, continuous sorghum cultivation served as the control, and three crop rotation systems were established: sorghum-potato-sorghum (SPS), sorghum-common vetch-sorghum (SVS), and sorghum-green pea shoots-sorghum (SGS) as experimental treatments, systematically investigating the effects of diversified crop rotation on soil nutrients, microbial community structure, and ecological functions in the sorghum rhizosphere. The diversified crop rotations designed in this study systematically improved the health of the soil in the sorghum rhizosphere through a cascade effect of “nutrient enhancement—community reshaping—functional differentiation”. Crop rotation not only increased soil organic matter and available nutrient content but also reshaped the structure of bacterial and fungal communities, enriched functional groups associated with carbon and nitrogen cycles, and enhanced the complexity and connectivity of microbial co-occurrence networks. These findings suggest that the design of crop rotation systems should fully consider the combination of crop functional types to better leverage the ecological advantages of different rotation patterns.

This study provides a scientific basis for optimizing crop rotation systems in Guizhou’s core sorghum-producing regions and offers theoretical support for alleviating continuous cropping obstacles and building healthy soils through ecological regulation. However, this study is based solely on short-term field experiments spanning two crop rotation cycles, and microbial function predictions rely on database annotations; their actual metabolic activity requires further verification. Future research should combine long-term field experiments with multi-omics technologies to deeply analyze the molecular mechanisms by which crop rotation drives the expression of soil microbial functions. It should also explore the development of beneficial functional microorganisms into specialized microbial inoculants, thereby providing more precise scientific and technological support for sustainable agricultural development.

## Data Availability

Raw data have been deposited in the National Center for Biotechnology Information (NCBI) under BioProject accession number PRJNA1544411 (https://www.ncbi.nlm.nih.gov/bioproject/PRJNA1544411).
